# Lipoma in the pronator quadratus: A case report

**DOI:** 10.1097/MD.0000000000020248

**Published:** 2020-05-22

**Authors:** Heng Tian, Wen-rui Qu, Junbo Pan, Zhe Zhu, Jun Liu, Rui Li

**Affiliations:** a Hand Surgery, the Second Hospital of Jilin University, No. 218, Lane Ziqiang, Nanguan District, Changchun City, Jilin; b Hand and Foot Surgery, the Affiliated Hospital of Yangzhou University, No. 368, Hanjiang Middle Road, Yangzhou City, Jiangsu, PR China.

**Keywords:** diagnosis, intramuscular lipoma, treatment

## Abstract

**Rationale:**

Lipomas are common benign tumors, constituting 16% of soft tissue mesenchymal tumors. They usually occur under the skin or in the large muscles of the thigh, shoulder, or upper arm. There are few reported cases of lipomas located in the forearm and hand muscles, accounting for less than 1% of all lipomas.

**Patient concerns:**

A 47-year old man presented with a history of swelling and accompanying pain in the left wrist for 2 years.

**Diagnoses:**

The patient was diagnosed with intramuscular lipoma in the pronator quadratus.

**Interventions:**

The mass was resected completely with wide-awake technique.

**Outcomes:**

The patient was followed up for 2 years with no recurrence. The symptoms of swelling and pain resolved within 3 weeks post-surgery, and there was no clear abnormality in wrist and finger movement and sensation. A satisfactory outcome was achieved.

**Lessons:**

Intramuscular lipoma in the pronator quadratus is a rare benign tumor which should be distinguished from malignant tumors. Especially for patients with carpal tunnel syndrome presenting with wrist swelling, ultrasound, computed tomography, or magnetic resonance imaging can be used to assess deep tissue masses.

## 1 Introduction

Lipomas are common benign tumors, constituting 16% of all soft tissue mesenchymal tumors.^[1]^ They usually occur under the skin or in the large muscles of the thigh, shoulder, or upper arm. There are rarely reported cases of lipomas located in the forearm and hand muscles,^[1,2]^ accounting for less than 1% of all lipomas. In this paper, we present the case of a lipoma in the pronator quadratus. No recurrence was observed in this case in 2 years of follow-up.

## 2 Case presentation

**Figure 1:**
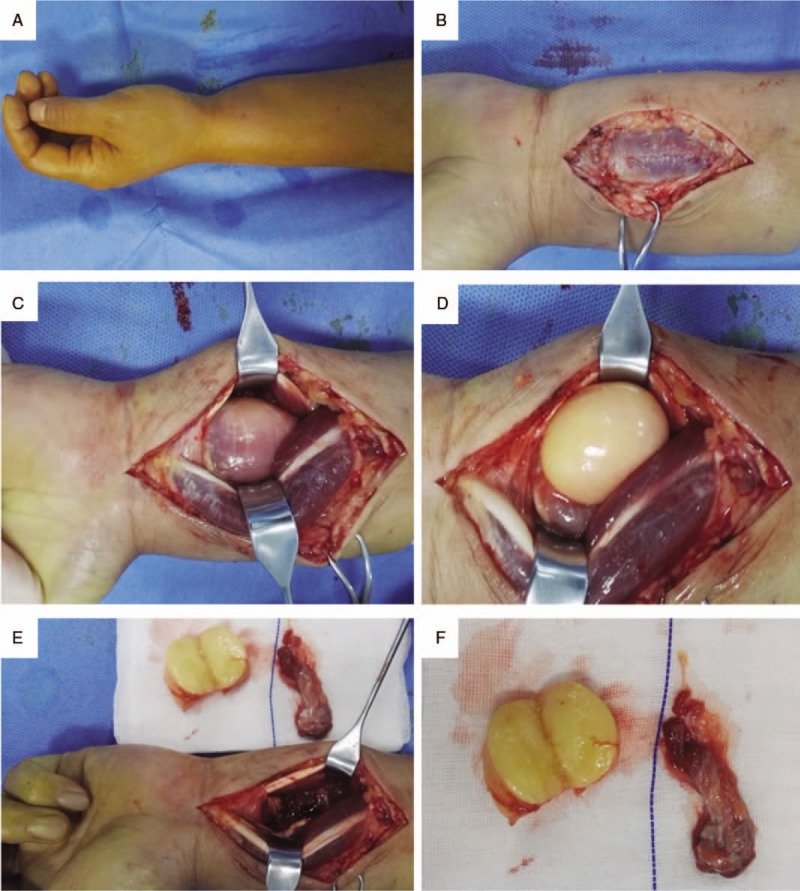
Appearance of the lipoma. A. Preoperative lateral view shows an intumescence at the distal end of the left forearm; B–E The mass and surrounding sarcolemma were revealed and completely removed layer by layer; F. Appearance of the postoperative tumor section and resected suspicious muscle bundle and sarcolemma.

A 47-year-old man presented with a 2-year history of swelling and accompanying pain in the left wrist, but no paresthesia. Massage and infrared treatment had resulted in no obvious improvement in these symptoms. Ultrasound examination revealed a 4.0 cm × 3.0 cm subcutaneous hypoechoic mass with no blood flow. Physical examination indicated no swelling of the superficial lymph nodes. There was an intumescence at the distal end of the left forearm, with no dark purple skin (Fig. 1A). On palpation, the local, diffused soft mass had an unclear boundary and normal skin temperature, was nonpulsatile, and did not move in different positions. Tinel sign was negative at the wrist.

Wide-awake technique^[3]^ was used for mass resection: a 6-cm arc-shaped incision was made at the ulnar edge of the long palmar muscle of the distal forearm, followed by peeling back the skin and subcutaneous tissues (Fig. 1B). No subcutaneous edema was observed. There was no obvious inflammation in the deep fascia and sarcolemma. Local intumescence was obvious, and no enclosed mass was felt in the flexor tendons or muscles. Subsequently, the seromuscular layers were exposed, and there was no synovial hyperplasia or other inflammatory changes in the tendon. After moving the flexor tendons, a yellowish-white mass wrapped with muscle fibers was observed on the lunar side of the pronator quadratus (Fig. 1C). The muscle fibers were bluntly peeled, and a 4.0 cm × 3.0 cm oval, yellowish-white, soft mass with an intact capsule emerged (Fig. 1D). The mass was not pedicled, and was not associated with the wrist capsule, joint cavity, ulnar nerve vascular bundle, or median nerve. The mass was completely separated from the capsule and the peripheral muscle bundles and sarcolemma were removed (Fig. 1E). After fully stopping the bleeding, the incision was rinsed and closed layer by layer. The mass was dissected after operation, and the section was composed of homogenous, yellow fine adipose tissues, with no clear infiltration, fibrosis, thrombosis, or necrosis of the muscle bundles (Fig. 1F). Pathological examination indicated a lipoma with no pathological changes in the muscle bundle and sarcolemma.

The resection was completed on an outpatient basis, and the patient went home immediately after the procedure. No antibiotic or pain medications were required. The symptoms of swelling and pain were resolved within 3 weeks post-surgery, and there was no clear abnormality in wrist and finger movement and sensation. There was no recurrence in 2 years of follow-up.

## 3 Discussion

A lipoma is the most common benign soft tissue tumor arising from mesenchymal tissues and is composed of mature adipose cells. Lipomas can occur at any age, but are most often seen in the 40 to 70 year old population, without gender preference.^[4,5]^ Lipomas are superficial, often involving subcutaneous adipose tissue, and deep lipomas can occur under the fascia. Intramuscular lipomas (IML) account for less than 1% of all lipomas.^[6]^ In 1853, Paget reported the first case of IML, followed by several reports of IMLs mostly located in the thigh, shoulders, and upper arm.^[7–10]^ IMLs of the forearm and hand are relatively rare, and have been reported in the thenar muscle and the thumb.^[1,11,12]^

As with other types of lipomas, the pathogenesis and infiltration mechanism of IMLs are unclear, and they may be solid tumors that originate directly from pluripotent mesenchymal cells. They are also thought to be associated with uncontrolled adipose growth caused by trauma, chronic irritation, obesity, developmental disorders, endocrine and metabolic disorders, or genetic factors. In addition, Ramos-Pascua et al^[13]^ found that two-thirds of patients with lipomas are overweight or obese, so the occurrence of IML may be related to increased body mass index. Mori believed that the occurrence of IML may be related to local neurogenic or myogenic diseases. He found that selective muscle fiber atrophy or degenerative changes often occur in IMLs, most of which are invasive. This infiltration is thought to relate to abnormally elevated levels of mobility group proteins.^[14]^ Fibrous atrophy is not limited to the area of fatty infiltration but can also be found in peripheral muscle fibers involved with the tumors.

IMLs often manifest as an asymptomatic, slow-growing mass or swelling with no obvious mass. The clinical manifestations are mainly determined by the position of the tumor growth. When the tumor is large enough to invade the muscle, limitation of motion may appear. When the peripheral nerve is pressed, it may cause paresthesia but generally does not cause pain.^[10]^ When an IML is located in the posterior pharyngeal space, the patient may develop difficulties in swallowing and speech due to recurrent laryngeal nerve and supra-larynx nerve compression.^[15]^ When an IML is located under the tongue, it may cause irritation and difficulties in swallowing and speech.^[16,17]^ An IML in the chest may even be misdiagnosed as breast cancer.^[18]^ Therefore, intramuscular lipomas are often misdiagnosed as malignant tumors. An IML in the pronator quadratus, as in this case, could also cause a disorder of forearm rotation by compression of the terminal branch of the anterior interosseous nerve, or produce carpal tunnel syndrome due to traction of the median nerve.

Imaging examination is the most important tool for the diagnosis of IML. Ultrasound is the first choice for its convenience, speed, and low cost. It can dynamically show the relationship between the location and depth of the tumor and the adjacent tissue. Under ultrasound, IMLs appear as intramuscular echogenic masses with clear borders and no blood flow signals, in which the echo intensity is higher than that of the muscle but similar to subcutaneous adipose tissues. The tumors encircled by muscle fibers can be heterogeneous and have internal visible streaks. Computed tomography (CT) and magnetic resonance imaging (MRI) can more clearly reveal the integrity of the capsule, the fat content in the tumor, the degree of fat and muscle, and the thickness and distribution of fiber intervals. Intramuscular low-density masses can be seen on CT; both high T1 and T2-weighted signals are observed on MRI. Fat suppression MRI sequencing is an available approach to comprehensively and clearly assess peritumoral edema and the degree and range of tumor invasion to the surrounding muscle tissues. It is also useful in identifying other tumors, and especially malignant tumors.^[19]^ In this case, the initial diagnosis was only based on the ultrasound results, and no further examination was performed to re-confirm the nature and location of the tumor. In general, we would caution implementing a surgical exploration, by which the tumor is prone to be misdiagnosed or missed, without thorough diagnostic imaging.

It is not difficult to diagnose IML based on symptoms, signs, and imaging examinations. However, most IMLs are invasive, and need to be differentiated from muscle spasm, hemangioma, liposarcoma, fibromyositis, and fibrosarcoma.^[20]^ Histopathological examination is the main approach to distinguish IML from well-differentiated liposarcoma. IMLs manifest with mature and single-celled adipocytes of equal size and uniformity, with occasional muscle fiber bulbs. The adipocyte nuclei are small, flat, and located peripherally, without cellular atypia, mitotic activity, polykaryon, or lipoblasts. In contrast, liposarcoma is a mixture of atypical cells or vacuolarized adipocytes with spindle-shaped fibroblast-like cells, often located in many thick intervals, with vascular components of varying sizes.^[21]^

Generally, asymptomatic IMLs can be assigned observation status, and the recurrence rate after surgical removal is less than 5%.^[2]^ IMLs can be divided into infiltrative, well-defined and mixed type. For well-defined masses, marginal resection is preferred. In infiltrating masses, muscle fiber bundles pass through the tumor, and a small number of IMLs are enlarged through other structures or surroundings, which necessitates expanded surgical resection. If complete resection is not possible or complete resection can lead to severe dysfunction, partial resection may be adequate. When the origin of the mass cannot be determined, but there is a secondary infiltration of adjacent muscles, a small amount of surrounding tissue can be removed.^[19]^ When it is difficult to make a judgment, preoperative puncture biopsy for pathology examination can be used to distinguish from other malignant tumors such as low-grade liposarcoma, potentially avoiding unnecessary expanded resection. The excised intramuscular lipoma is composed of localized, uniform yellow adipose tissues, which is a somewhat brownish color, usually without a capsule but with a soft texture. There are usually no signs of necrosis or bleeding visible to the naked eye.^[21]^

## 4 Conclusion

In summary, IML in the pronator quadratus is a rare benign tumor which should be distinguished from malignant tumors. Especially for patients with carpal tunnel syndrome presenting with wrist swelling, ultrasound, CT or MRI can be used to differentiate IMLs from other deep tissue masses.^[22,23]^

## Author contributions

All authors substantially contributed to the manuscript. TH and QWR designed the study. PJB and LJ performed the literature review, extraction of the data, and analysis of the pooled data. TH and ZZ performed surgical resection and perioperative care. LR reviewed and revised the manuscript. All authors read and approved the final manuscript.

**Data curation:** Wenrui Qu, Zhe Zhu.

**Formal analysis:** Wenrui Qu.

**Investigation:** Heng Tian, Wenrui Qu, Zhe Zhu.

**Project administration:** Rui Li.

**Resources:** Zhe Zhu, Jun Liu.

**Supervision:** Jun Liu.

**Writing – original draft:** Heng Tian, Junbo Pan.

**Writing – review & editing:** Rui Li.
